# Posterior Corneal Lamellar Detachment after Phacoemulsification in a Case of Anterior Lamellar Keratoplasty

**DOI:** 10.18502/jovr.v14i3.4797

**Published:** 2019-07-18

**Authors:** Deepika Dhingra, Chintan Malhotra, Vaneet Jakhar, Vikash Rohilla, Arun Kumar Jain

**Affiliations:** Advanced Eye Centre, Department of Ophthalmology, Postgraduate Institute of Medical Education and Research, Chandigarh, India

##  PRESENTATION

**Figure 1:**
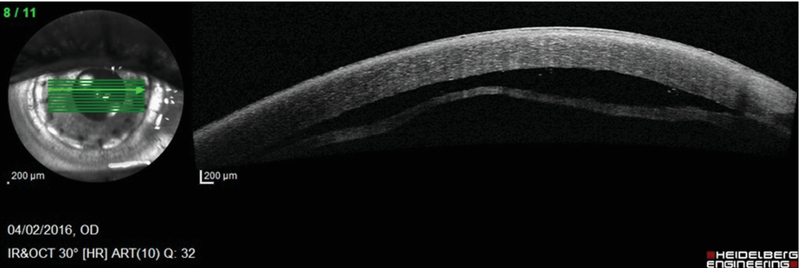
Anterior segment optical coherence tomography showing separation of anterior lamellar graft and posterior lamella.

**Figure 2:**
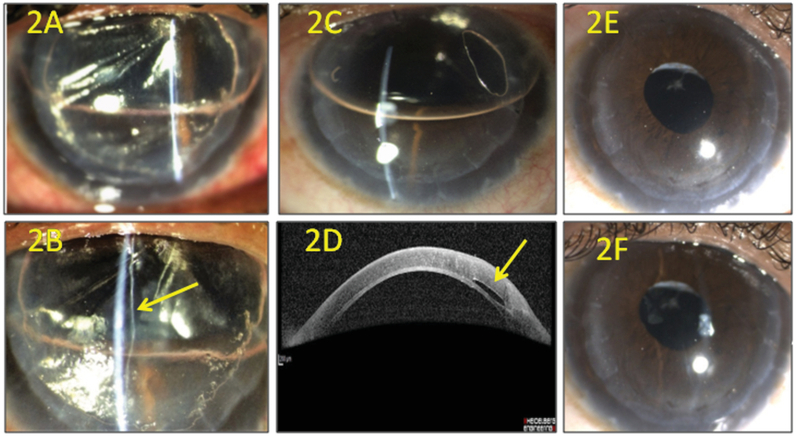
(a) Day 1 after C3F8 gas tamponade, with gas bubble in anterior chamber (AC) and gas bubble entrapment between the lamellae. (b) Magnified slit view shows entrapped gas bubble in the potential space between anterior and posterior lamellae. (c) Immediate reattachment of anterior and posterior lamellae after slit-lamp drainage of entrapped gas bubble with only a small residual gas bubble. (d) Anterior segment optical coherence tomography after gas bubble drainage shows reattachment of lamellae and a small residual gas bubble in the interface. (e and f) Clear graft with well-placed intraocular lens at the 18-month follow-up.

A 39-year-old female with a history of automated anterior lamellar keratoplasty for a nebulomacular corneal opacity in the right eye presented with a gradually progressive decrease in vision two years ago. Presenting visual acuity (PVA) was 20/200, and a grade 3 nuclear cataract was noted.^[1]^ The patient underwent coaxial phacoemulsification with posterior chamber intraocular lens implantation through a 2.2 mm clear corneal incision under peribulbar anesthesia. During the hydration of the incisions, the main port incision was noted to be leaky. A possible disruption in the graft–host lamellar interface was suspected intraoperatively, due to the inner lip of the main port incision opening in the lamellar interface. The incision was closed with two interrupted sutures of 10-0 nylon, and the globe was well-pressurized by forming the anterior chamber (AC) with balanced salt solution followed by the injection of an air bubble. On the postoperative day 1, PVA was 20/400 with mild graft edema, AC was well formed, and the air bubble was in the upper third of the AC. On the follow-up at two weeks, the patient reported further diminution of vision for seven days. PVA had decreased to “hand motion” close to the face, and there was significant graft edema along with    presence of fluid between the anterior lamellar graft and posterior lamella leading to the formation of double AC, as observed on anterior segment optical coherence tomography (OCT) (Heidelberg Engineering GmbH, Heidelberg, Germany) [Figure 1]. Intracameral 14% perfluoropropane (C3F8) gas was injected, and supine posture was advised for one day. On the subsequent day, PVA was “counting fingers” at 1 m. The gas bubble was partially in the AC and partially entrapped between the corneal lamellae [Figures 2(a) and (b)]. The interface gas bubble was drained on the slit-lamp with a 26-gauge needle entering through the mid-peripheral cornea. This immediately led to the reattachment of the lamellae (supplemental video) and improvement in graft clarity with only a small residual interface gas bubble away from the visual axis [Figures 2(c) and (d)]. A final uncorrected VA of 20/60 and best-corrected VA of 20/40 with a refraction of +0.5 DC × 165${}^{\text{o}}$ was achieved. The graft maintained its clarity till the last follow-up, 18 months after phacoemulsification [Figures 2(e) and (f)].

##  DISCUSSION

Even after complete healing, the tensile strength of corneal wound is less than that of the intact cornea. The lamellar interface heals with weak adhesions as observed clinically by the ease of lifting of LASIK flap even years after surgery.^[2,3]^ The probable reason for lamellar detachment occurring twice in a short span, in this case, could be the clear corneal incision traversing the lamellar interface, leading to the opening of this potential space by the continuous infusion of fluid from the AC during phacoemulsification and in the early post-operative period and by the C3F8 bubble later on. Performing phacoemulsification through a scleral tunnel instead of a clear corneal incision may help avoid this complication.

##  Financial Support and Sponsorship

Nil.

##  Conflicts of Interest

There is no conflict of intrest.
